# Combined Spectroscopic and Calorimetric Studies to Reveal Absorption Mechanisms and Conformational Changes of Protein on Nanoporous Biomaterials

**DOI:** 10.3390/ijms160817289

**Published:** 2015-07-29

**Authors:** Saharnaz Ahmadi, Maryam Farokhi, Parisa Padidar, Mojtaba Falahati

**Affiliations:** 1 Department of Biology, Science and Research Branch, Islamic Azad University, Tehran P. O. Box: 1477893855, Iran; E-Mail: saharnazahmady@gmail.com; 2 Department of Nanotechnology and Advanced materials, Materials and Energy Research Center, Tehran P. O. Box: 31787316, Iran; E-Mail: farokhi.maryam@gmail.com; 3 Department of Nanotechnology, Faculty of Advance Science and Technology, Islamic Azad University of Pharmaceutical Sciences (IAUPS), Tehran P. O. Box: 193956466, Iran; E-Mail: baran7959@yahoo.com

**Keywords:** protein, nanoporous, immobilization, BLG-B, calorimetry

## Abstract

In this study the effect of surface modification of mesoporous silica nanoparticles (MSNs) on its adsorption capacities and protein stability after immobilization of beta-lactoglobulin B (BLG-B) was investigated. For this purpose, non-functionalized (KIT-6) and aminopropyl-functionalized cubic *Ia3d* mesoporous silica ([*n*-PrNH_2_-KIT-6]) nanoparticles were used as nanoporous supports. Aminopropyl-functionalized mesoporous nanoparticles exhibited more potential candidates for BLG-B adsorption and minimum BLG leaching than non-functionalized nanoparticles. It was observed that the amount of adsorbed BLG is dependent on the initial BLG concentration for both KIT-6 and [*n-*PrNH_2_-KIT-6] mesoporous nanoparticles. Also larger amounts of BLG-B on KIT-6 was immobilized upon raising the temperature of the medium from 4 to 55 °C while such increase was undetectable in the case of immobilization of BLG-B on the [*n*-PrNH_2_-KIT-6]. At temperatures above 55 °C the amounts of adsorbed BLG on both studied nanomaterials decreased significantly. By Differential scanning calorimetry or DSC analysis the heterogeneity of the protein solution and increase in *Tm* may indicate that immobilization of BLG-B onto the modified KIT-6 results in higher thermal stability compared to unmodified one. The obtained results provide several crucial factors in determining the mechanism(s) of protein adsorption and stability on the nanostructured solid supports and the development of engineered nano-biomaterials for controlled drug-delivery systems and biomimetic interfaces for the immobilization of living cells.

## 1. Introduction

The design of new nanoporous biomaterials with appropriate properties for the efficient immobilization of bio-macromolecules has great potential in different areas, such as biotechnology, biocatalysts, protein-delivery systems and biosensors [1]. The main strategy is based on the synthesis and development of functional biocompatible host supports with suitable interfacial structures assuring firm attachment of bio-macromolecules while preserving their native structures and corresponding functions as much as possible. Therefore in order to enhance the efficiency of native biological molecules immobilization on the solid supports, it is highly recommended to develop nanostructured matrices with large surfaces, high porosity and stabilities.

Ideal immobilization methods would lead to protein stability over long periods of time and minimize protein conformational change and leaching. Nanotechnology progress in bio-analytical applications have created inorganic phases with excellent and suitable interfaces with nanostructured, highly ordered pore size distributions and high surface areas such as mesoporous silica nanoparticles (MPNs) for immobilization of a wide range of proteins [1,2,3]. After the discovery of MPNs in 1992 by Beck *et al.*, their potential application in protein confinement was developed in 1996 by Diaz and Balkus [4,5]. In recent years a wide range of interest has been directed to nanoporous materials for immobilization of native structure proteins [6,7,8,9,10,11]. Their relatively large pore diameters (2–40 nm), narrow pore size distribution, large pore volumes (*ca.* 1.5 cm^3^·g^−1^) and a high surface area (up to 1200 m^2^·g^−1^) enable their use as great candidates to match the size of the wide range of biologically active molecules [5,6,10]. In addition, they present an extremely stable structure, chemically, mechanically and thermally, with low toxicities owing to their inorganic silicate network that makes them ideal candidates for nanobiotechnology applications [12,13,14,15,16,17]. However, weak hydrophilic interactions between the active molecules and nanoporous surfaces result in frequent leaching of the adsorbed bio-macromolecules [18].

Therefore, defined strategies such as decreasing the sizes of pore openings and functionalization of the inorganic surfaces with organic moieties can be applied to protect the confined biomolecules from leaching [19,20,21,22,23,24,25,26,27]. Functionalization of the nanoporous surface is a potentially more appealing route for protein immobilization due to the presence of higher adsorption affinity for adsorbed bio-macromolecules [20,21,22,23,24,25,26,27]. Therefore, the weak interactions between matrix and biomolecules can be strengthened by the functionalization of mesoporous nanoparticle surfaces and absorption of the biological macromolecules on such “hybrid nanocomposites”. Nanoporous geometry plays a key role in stabilizing properties of MPNs and preventing leaching [28,29,30,31]. Proteins can be adsorbed inside the matrix of MPNs using different approaches, including physical adsorption, encapsulation and chemical binding [32,33]. However, most reports have so far been dedicated to the synthesis, characterization, and functionalization of different types of MPNs [34,35,36,37].

Relatively little effort has been oriented to the effect of functionalization, initial protein concentration and raising the temperature on the efficacy of protein adsorption and its stability [38]. In this study special emphasis is placed on the usage of the new type of MPNs called KIT-6, which consists of a 3D cubic structure, and *Ia3d* symmetry with the interpenetrating bicontinuous channels [39].

Beta-lactoglobulin also was used as a model which is a small dimeric globular protein with molecular mass of ~18 kDa, hydrodynamic radius of 2 nm and isoelectric point of 4.5. In native BLG-B, two disulphide bridges (Cys66-Cys160 and Cys106-Cys119) and the free thiol group (Cys121) are placed deeply in a hydrophobic core of globulin [40,41,42,43,44].

This research, compared to previous studies [45,46], is focused mainly on the influence of aminopropyl-functionalization of mesoporous nanoparticles, BLG-B concentration and raising temperature on the amount of BLG adsorption on the KIT-6 solid supports. Also the DSC experiments were designed for the performance of analytical (homogeneous/heterogeneous protein population) and numerical calculations of protein stability. By comparison, a limited amount of research has been conducted to characterize the heterogeneity of matrix-absorbed proteins. In this report we directly probe the relationship between the protein absorption characteristics and the heterogeneity in the protein’s affinity and binding constant. The obtained data can provide useful guidance for designing appropriate nanostructured supports for application in materials-delivery systems.

## 2. Results and Discussion

The cubic mesoporous silica nanomaterials (KIT-6 and [*n*-PrNH_2_-KIT-6]) were fully characterized by X-ray diffraction (XRD), transmission electron microscopy (TEM), N_2_ adsorption-desorption isotherm, FT-IR spectroscopy, and thermal gravimetric analysis (TGA) as reported in previous papers [45,46].

### 2.1. Effect of Aminopropyl-Functionalization of Mesoporous Nanoparticles on the Amount of Adsorbed BLG

The surface of nanoporous silica was modified for enhancing protein absorption. Table 1 shows the amount of protein adsorbed and leached on/from the non-functionalized and aminopropyl-functionalized solids supports. It was observed that the KIT-6 support adsorbed 28.1% of the BLG-B, while our previously data demonstrated higher protein adsorption (63.8%) occurred on functionalized KIT-6 [46]. This large amount of loading of BLG-B in the modified matrix revealed that the protein molecules were not only confined inside the support channels but also there must be a substantial fraction of protein adsorbed on the external surface of KIT-6 [47]. Adsorption of molecule to the solid support is a consequence of favorable interactions. Proteins on the functionalized support surface form more energetically driven bonds (physisorption or chemisorption) than non-functionalized ones due to the presence of partner atoms.

However, leaching of 21.5% was measured for BLG-B-KIT-6 after stirring in a buffer solution for 2 h, while as no leaching was observed for the BLG-B-[*n*-PrNH_2_-KIT-6] system [46].

**Figure 1:**
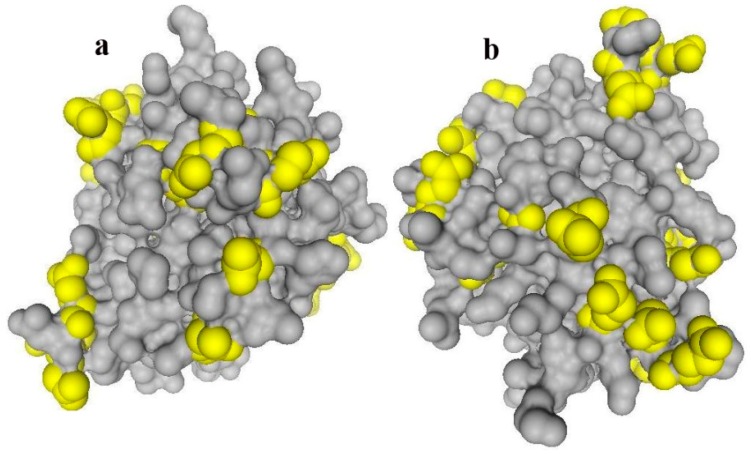
Distribution of carboxylic residues (yellow) on the BLG surface (PDB number: 3BLG) from two rotational views (**a**,**b**). This figure is generated by using the software of Swiss-PdbViewer version of 4.1.

Under the experimental conditions (pH = 7.8), negative charges were dominant on the BLG surface (protein data bank or PDB no: 3BLG, isoelectric points (IEP) = 4.5) (Figure 1). However, the IEP of [*n-*PrNH_2_-KIT-6] increased from 2.5 to 9.5 owing to the presence of amine groups (–NH_3_^+^), while for pure KIT-6 with silica IEP of 2.5, negative charges are distributed on the solid support surface. Therefore, there is an electrostatic repulsion between BLG-B molecules and the silica surfaces of the non-functionalized mesoporous nanomaterials, while amine functionalization facilitates the formation of electrostatic attraction between BLG and modified support surface. Hence repulsion or attraction forces would be responsible for the main differences of loading and leaching of protein on nanoporous supports.

**Table 1:** The percentage of adsorption and leaching of BLG-B on/from the KIT-6 and [*n*-PrNH_2_-KIT-6].

Supports	Pore Size (nm)	BLG Adsorbed (%)	BLG Leached (%)
KIT-6	7.2	28.1	21.5
[*n*-PrNH_2_-KIT-6] [46]	6.5	63.8	0

### 2.2. Effect of Initial Concentration of BLG Solution on the Amount of Protein Immobilization on the KIT-6 and [n-PrNH_2_-KIT-6]

**Figure 2:**
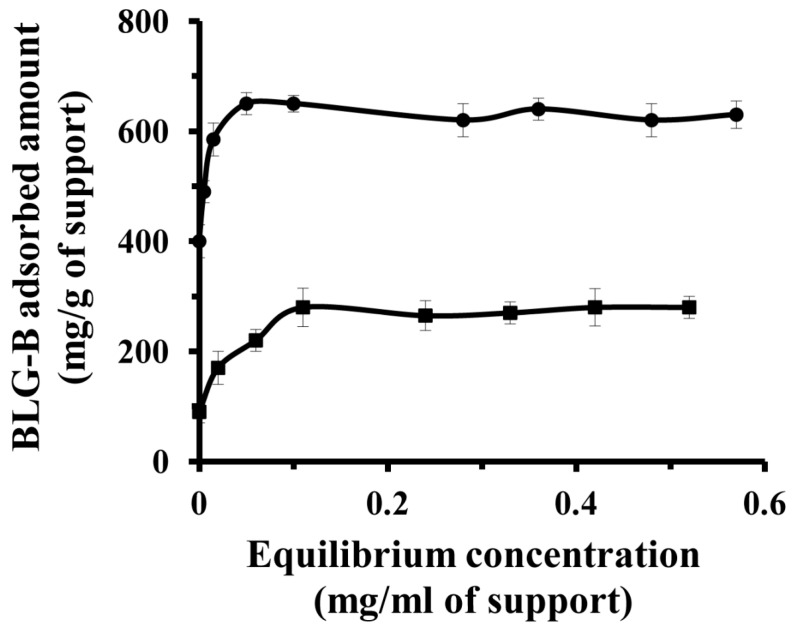
Effect of initial protein concentration on the adsorption of BLG-B on KIT-6 (■) and [*n*-PrNH_2_-KIT-6] (●) mesoporous silica nanomaterials.

Ideally, it is desirable to use an immobilization support that maximizes the stability of all the confined proteins and thereby minimizes the amount of inactive and denatured protein. In order to consider the contribution of physical and chemical interactions on immobilization of BLG-B on the different kind of nanoporous supports (non-functionalized and aminopropyl-functionalized), the adsorption isotherms were measured. Figure 2 demonstrates single molecule layer adsorption based on Langmuir equation and a different behavior of the immobilization of BLG-B on the [*n*-PrNH_2_-KIT-6] in respect to KIT-6. The difference between absorption content reveals a higher affinity of amine-solid surfaces to BLG-B in comparison to KIT-6.

The Langmuir equation relates the fractional coverage of host solid surface to the concentration of protein in solution.

(1) $$q=\frac{q\text{max}\times c}{Kd+c}$$

Here *K_d_* is the dissociation constant of the BLG-B–support complex, *c* is the BLG-B concentration, *q*_max_ is the maximal amount of BLG-B adsorbed on the supports, and *q* is the amount of BLG-B adsorbed on the host solid. The dissociation constant (*K_d_*) and the maximal amount of BLG-B immobilized on the solid support (*q*_max_) were calculated to be 0.002 mg/mL and 665 mg·g^−1^ for BLG-B-[*n*-PrNH_2_-KIT-6] and 0.014 mg/mL and 285 mg·g^−1^ for BLG-KIT-6, respectively. This increment rate of adsorption of BLG-B on [*n*-PrNH_2_-KIT-6] relative to KIT-6 is corresponding to presence of supporting linkage (electrostatic attractions) in addition to hydrophobic and hydrogen bonds, which are common interactions in adsorption of proteins on the non-functionalized mesoporous nanoparticles. The dissociation constants values also revealed that the dominant network linkages between BLG-B and [*n*-PrNH_2_-KIT-6] were considerably stronger than that of KIT-6. In addition, the shape of the isotherm curves exhibited a slower adsorption rate of BLG onto the KIT-6 surfaces compared to [*n*-PrNH_2_-KIT-6]. These results reveal that immobilization of protein on the nanoporous matrixes was strongly dependent on the bulk concentration of the BLG-B solutions; the adsorbed amount on solid supports gradually increased with the raising protein solution concentration. The adsorption isotherms (Figure 2) further indicated the affinity of BLG-B for the different surfaces; [*n*-PrNH_2_-KIT-6] showed higher adsorption capacity for increasing concentrations of BLG-B solutions.

The adsorption of protein on nanoporous solid supports may be defined as diffusion of protein molecules to the nanoporous materials and finally binding to the surfaces of support. In this study, electrostatic interactions are the dominant and crucial forces in the immobilization of BLG-B on the amine-functionalized nanoporous support relative to non-functionalized one.

### 2.3. Effect of Temperature on the Amount of BLG-B Adsorption on the KIT-6 and [n-PrNH_2_-KIT-6]

**Figure 3:**
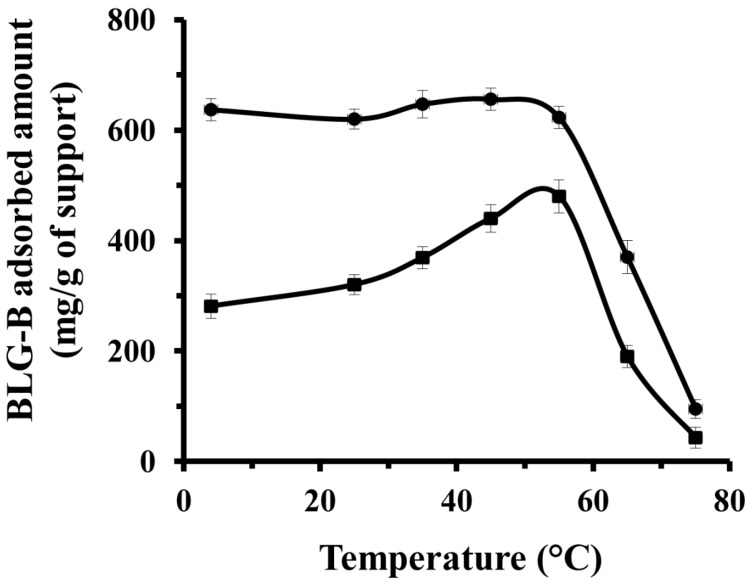
Effect of temperature on the adsorption amounts of BLG-B on KIT-6 (■) and [*n*-PrNH_2_-KIT-6] (●) mesoporous silica nanomaterials.

Heat stress can induce conformational change of proteins and therefore influence their adsorption behaviors onto the solid supports. Hence, the effect of elevation of medium temperature (from 4 to 75 °C) on immobilization of BLG-B onto the modified and unmodified KIT-6 was investigated. Figure 3 shows the immobilized content of BLG-B on the non-functionalized KIT-6 increases dramatically with raising temperature from 4 to 55 °C (from 281 mg/g of support at 4 °C to 480 mg/g of support at 55 °C), while varying temperature does not bring any changes in immobilization amount of BLG-B on the [*n*-PrNH_2_-KIT-6]. These observations may indicate that the BLG-B concentrations selected for this experiment did not saturate the surface of KIT-6 and that the interfaces were probably exposed after raising temperature. 

These data may also provide information about the heterogeneity in the absorbed protein population onto KIT-6 support due to randomly oriented attachment of proteins. Considering our results, it can be concluded that these factors favored higher levels of BLG-B adsorption on to the KIT-6 matrix: heterogeneity, wettability and surface energy, which characterized the differences between non-functionalized and functionalized solid supports. In presence of amine functional groups, all these factors are changed and result in more adsorption capacity for a functionalized matrix. It has been reported that the diffusion and absorption of the protein in the inner walls of the nanoporous support is combined with an endothermic process to rearrange the linkages between solid nanomaterials and previously immobilized protein [38]. Therefore breaking of interactions between matrix and absorbed BLG-B after raising temperature and corresponding rearrangement of proteins and additional free available space for immobilization of protein is another key factor responsible for the larger amount of adsorbed BLG-B on KIT-6 and production of heterogeneous protein population. This rearrangement cannot be just limited to the inner part of nanopores but also can occur for the surface of pores and their absorbed proteins. In contrast, in the case of BLG-B immobilized on [*n*-PrNH_2_-KIT-6] no significant increment rate in adsorption was observed by varying the temperature.

This observation is attributed to the steric hindrances of the surface and inner part of nanoporous solid supports because of large amounts of absorbed BLG-B in aminopropyl-functionalized mesoporous nanomaterials. Therefore affinity of BLG-B to [*n*-PrNH_2_-KIT-6] is close to maximal and the additional binding is stopped. However, the amount of adsorbed BLG on both kinds of non-functionalized and functionalized solid supports was reduced by raising temperature above 55 °C. It has been reported that heat stress over 55 °C shifts the conformational changes (monomer ↔ dimmer) of BLG in favor of monomers. Raising temperature to 55 °C induces a slight conformational change of the monomers, which has led to a “molten globule state”. Therefore native-like secondary structure of BLG remains intact, but the tight packing of the native tertiary structure is undergoing conformational changes. Above 65 °C, larger secondary and tertiary structural change occurs and leads to thiol/disulphide exchange mechanism and corresponding unfolding and exposure of hydrophobic portions of the protein, promoting aggregation of the protein with a hydrodynamic radius much larger than that of the pore diameter of matrix support [48,49,50,51,52].

### 2.4. DSC Measurements of Free and Immobilized BLG-B

**Figure 4:**
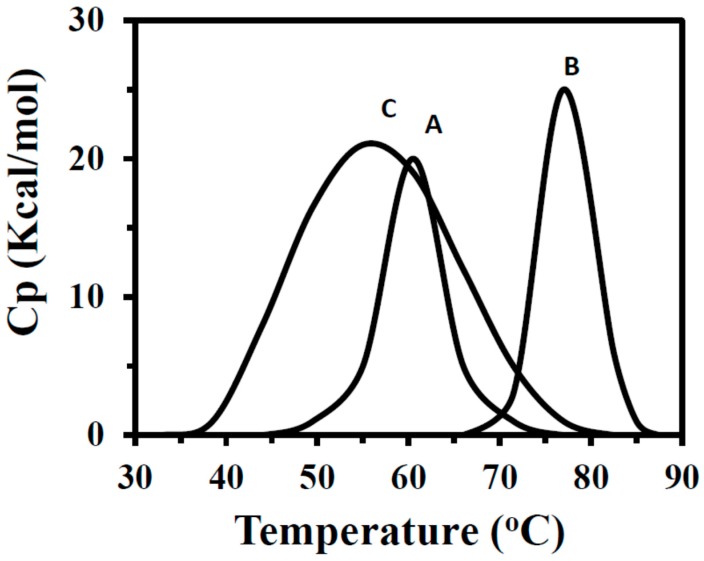
DSC profiles of free BLG (A), BLG-[*n*-PrNH_2_-KIT-6] (B) and BLG-B-KIT-6 (C).

Thermal unfolding processes in free and immobilized BLG-B were followed by DSC technique. Analysis of DSC profile of BLG-B-[*n*-PrNH_2_-KIT-6] demonstrated a sharper thermal profile (homogeneous population of BLG-B) with a 54.7 kcal/mol enthalpy value compared to that of the free BLG-B (with the value of 37.5 kcal/mol) and BLG-B-KIT-6 (with the value of 173.5 kcal/mol). The transition points (*Tm*) of the free and immobilized BLG-B onto the KIT-6 and [*n*-PrNH_2_-KIT-6] were 60.5, 55 and 77 °C, respectively (Figure 4, Table 2). The heterogeneity of protein population can be determined by the value of enthalpy.

**Table 2:** Typical DSC results of free and immobilized BLG.

Supports	*Tm* (°C)	*∆H°* (Kcal/mol)
**Free BLG**	60.5	37.5
**BLG-[*n*-PrNH_2_-KIT-6]**	77	54.7
**BLG-KIT-6**	55	173.5

Therefore, attachment of BLG-B molecules to the non-functionalized KIT-6 solid support yield a heterogeneous and unstable population of the protein (*∆T* = −5.5 °C) in comparison with free ones whereas for BLG-B-[*n*-PrNH_2_-KIT-6] all samples exhibit a significant stable and homogeneous population of attached protein (*∆T* = 16.5 °C).

Therefore, by comparison of *Tm* and *∆H*, it may be concluded that immobilization of BLG-B on [*n*-PrNH_2_-KIT-6] leads to more energetically favorable interactions relative to KIT-6 on protein structure. Calorimetric data therefore exhibited that a distribution of structurally-rate forming states of bound BLG-B are dominant in the attachment of protein onto the non-functionalized solid support.

DSC results suggest that the extent of heterogeneity is largely influenced by bound protein. It seems that variability in the surface chemical functional groups or the extent of protein conformational change may differ between the tested nanoporous solid supports. Apparent variation in the protein heterogeneity may also arise if the BLG-B adsorbs non-specifically to the nanoporous surface rather than to the inner matrix of channels binding sites.

Varying the host solid supports would most likely change protein activity and heterogeneity by modifying the density of proteins on the non-functionalized and functionalized solid supports.

It can be suggested that beyond an optimum surface density, the apparent protein attachment decreases owing to steric hindrance between neighboring proteins. In addition, it is possible that the extent of protein heterogeneity can also alter with protein surface coverage. At sufficiently high surface densities of immobilized protein the lateral protein-protein interactions may order the proteins so that they show more uniform binding. Other variables such as the binding behavior may depend on the physical characteristics of the nanoporous matrix such as the roughness of the solid support. The surface topology of underlying host support could influence the orientation of the attached proteins so that matrix-protein binding becomes more heterogeneous. With attachment of BLG-B on the aminopropyl-functionalized solid support, the highest structural stability and the most homogeneous binding affinities of bound protein were observed. These results suggest that the extent of heterogeneity in the protein adsorption is directly related to the degree of protein stability.

These differences in protein stability after immobilization onto the nanoporous solid supports may have been referred to the types of protein interaction and protein absorption.

## 3. Experimental Section

### 3.1. Materials

Bovine BLG-B was purchased from Sigma (St. Louis, MO, USA). Phosphate buffer (20 mM, pH 7.8) was used in all experiments described and all salts were purchased from Merck (Darmstadt, Germany).

### 3.2. Methods

#### 3.2.1. Synthesis and Characterization of Non-Functionalized (KIT-6) and Aminopropyl-Functionalized Nanoparticles ([*n*-PrNH_2_-KIT-6])

The non-functionalized and functionalized mesoporous silica nanoparticles (KIT-6 and [*n-*PrNH_2_-KIT-6]) were synthesized and fully characterized as described in our previous reports [45,46] by X-ray diffraction (XRD), transmission electron microscopy (TEM), FT-IR spectroscopy, Brunauer-Emmett-Teller and Barrett-Joyner-Halenda (BET & BJH) methods, thermal gravimetric analysis (TGA), Zeta-potential titrations, CHN elemental analysis and back titration.

#### 3.2.2. Immobilization of BLG-B on Non-Functionalized (KIT-6) and Aminopropyl-Functionalized Mesoporous Silica Nanoparticles ([*n*-PrNH_2_-KIT-6])

Ten mg of both mesoporous solid supports (KIT-6 and [*n-*PrNH_2_-KIT-6]) were added to BLG-B solution (20 mM phosphate buffer, pH 7.8) at 4 °C under stirring and after 24 h the supernatant was separated by centrifugation at 6000 rpm for 10 min. The amount of BLG-B immobilized on both non-functionalized and aminopropyl-functionalized solid supports was measured spectrophotometrically at 278 nm using the Beer-Lambert equation. Then washing steps (three times) were used to remove BLG-B molecules retained on the external surfaces of the solid supports from those adsorbed inside the nanoporous silica. Finally BLG-B-matrixes were air dried and stored at 4 °C for further experiments.

#### 3.2.3. Assessment of Leaching of BLG-B from Non-Functionalized (KIT-6) and Aminopropyl-Functionalized Nanoparticles ([*n*-PrNH_2_-KIT-6])

Leaching experiments were also performed to measure the amount of BLG-B releases from mesoporous silica nanaoparticles into the bulk solution. The immobilized BLG-B on both solid supports were resuspended by stirring in a 20 mM phosphate buffer pH = 7.8 for 2 h and Bradford assay was used to measure the amount of BLG-B leached from the solid nanoparticles.

#### 3.2.4. Effect of Initial BLG Concentration on the Amount of Protein Adsorption on Non-Functionalized (KIT-6) and Aminopropyl-Functionalized Nanoparticles ([*n*-PrNH_2_-KIT-6])

A series of standard BLG-B solutions with varying concentrations ranging from 1 to 12 mg/mL were stirred with 10 mg of [*n*-PrNH_2_-KIT-6] and KIT-6 at 4 °C for 24 h. Then the equilibrium concentration and adsorbed amount of BLG-B was measured spectrophotometrically at 278 nm using Beer-Lambert equation.

#### 3.2.5. Effect of Temperature on the Amount of BLG-B Adsorption on Non-Functionalized (KIT-6) and Aminopropyl-Functionalized Nanoparticles ([*n*-PrNH_2_-KIT-6])

Ten mg of aminopropyl-functionalized mesoporous silica [*n*-PrNH_2_-KIT-6] and KIT-6 was added to 10 mL of 1 mg/mL BLG-B solution and stirred for 24 h at different range temperature of 4–75 °C in 20 mM phosphate buffer at pH 7.8. The supernatant was separated from the solid nanoparticles by centrifugation at 6000 rpm for 10 min and the content of adsorbed BLG-B onto the both supports was then determined using UV adsorption at 278 nm.

#### 3.2.6. Calorimetric Study of Free and Immobilized BLG-B onto Mesoporous Silica Supports

DSC measurement of free and immobilized BLG-B (1 mg/mL) was done in an instrument (Scal-1 microcalorimeter, Moscow, Russia) equipped with a 0.324 mL cell at a heating scan rate of 2 °C/min. The values of *Tm* and Δ*H* were calculated from thermograms using CpCalc software (version 2.1) and by subtraction of the buffer baseline (blank buffer (phosphate buffer), KIT-6 and [*n*-PrNH_2_-KIT-6] solid supports) solutions from the samples.

## 4. Conclusions

Surface modification and charge distribution considerably affect the network interaction and stability of the protein–nanoporous support system. This fact is due to the presence of additional hydrophobic and hydrophilic interactions that are the main driving forces of the adsorption of proteins on the host materials.

The immobilization of BLG-B on non-functionalized and aminopropyl-functionalized KIT-6 nanoparticles was studied and the effect of functionalization, initial concentration of protein and temperature on the amount of absorbed BLG was reported. It was revealed that the functionalization of KIT-6 with aminopropyl groups leads to formation of more efficient linkage between BLG and support. KIT-6 functionalized with aminopropyl groups leads to a higher degree of BLG-B adsorption and lower leaching rates of BLG-B compared with KIT-6. This difference is attributed to the coexistence of both hydrophobic and hydrophilic sites on modified silica surface. A single molecule layer adsorption behavior for absorption of BLG-B on the KIT-6 and [*n*-PrNH_2_-KIT-6] mesoporous silica nanoparticles was observed. It was also revealed that the amount and binding of BLG-B on the aminopropyl-functionalized mesoporous nanoparticle was considerably greater than on the non-functionalized nanoporous support.

Raising temperature from 4 to 55 °C lead to a significant enhancement of the adsorbed BLG-B on the KIT-6 while absorbed BLG on the [*n*-PrNH_2_-KIT-6] was almost constant. This effect was found to be due to the saturation and steric hindrance of BLG-[*n*-PrNH_2_-KIT-6]. Above 55 °C the amount of adsorbed BLG-B on both nanoporous supports were decreased. This reduction in protein immobilization indicates conformational changes and formation of aggregates of BLG-B at higher temperatures. Although protein immobilization onto the KIT-6 solid support yielded heterogeneous populations of BLG-B, immobilization of BLG-B onto the aminopropyl-functionalized matrix did produce a uniform protein population.

Attachment of functional moieties to the surfaces of solid supports has great interest for various biomedical and biotechnical applications. However, it is crucial that the biological properties of the confined bio-macromolecules are preserved during the adsorption. Hence it is important to limit or even diminish nonspecific interactions of biologically active molecules to the surfaces of the nanostructured solid support. These findings may provide useful information about the structural changes, compactness and stability of proteins during immobilization processes.
